# MLST genotypes of *Campylobacter jejuni* isolated from broiler products, dairy cattle and human campylobacteriosis cases in Lithuania

**DOI:** 10.1186/s12879-017-2535-1

**Published:** 2017-06-15

**Authors:** Sigita Ramonaite, Egle Tamuleviciene, Thomas Alter, Neringa Kasnauskyte, Mindaugas Malakauskas

**Affiliations:** 1 Department of Food Safety and Quality, Faculty of Veterinary Medicine, Veterinary Academy, Lithuanian University of Health Sciences, A. Mickeviciaus st. 9, LT 44307 Kaunas, LT Lithuania; 2 Clinic of Children Diseases, Medicine Academy, Lithuanian University of Health Sciences, Kaunas, Lithuania; 3 Institute of Food Safety and Food Hygiene, Freie Universität Berlin, Berlin, Germany

**Keywords:** *Campylobacter jejuni*, Multilocus sequence typing (MLST), Dairy cattle, Broiler products, Human campylobacteriosis, Genetic diversity

## Abstract

**Background:**

*Campylobacter* (*C.*) *jejuni* is the leading cause of human campylobacteriosis worldwide. We performed a molecular epidemiological study to investigate the genetic relationship among *C. jejuni* strains isolated from human diarrhoeal patients, broiler products and dairy cattle in Lithuania.

**Methods:**

The *C. jejuni* isolates from human clinical cases, dairy cattle and broiler products were genotyped using multilocus sequence typing (MLST). Allele numbers for each housekeeping gene, sequence type (ST), and clonal complex (CC) were assigned by submitting the DNA sequences to the *C. jejuni* MLST database (http://pubmlst.org/campylobacter). Based on the obtained sequence data of the housekeeping genes a phylogenetic analysis of the strains was performed and a minimum spanning tree (MST) was calculated.

**Results:**

Among the 262 *C. jejuni* strains (consisting of 43 strains isolated from dairy cattle, 102 strains isolated from broiler products and 117 clinical human *C. jejuni* strains), 82 different MLST sequence types and 22 clonal complexes were identified. Clonal complexes CC21 and CC353 predominated among the *C. jejuni* strains. On ST-level, five sequence types (ST-5, ST-21, ST-50, ST-464 and ST-6410) were dominating and these five STs accounted for 35.9% (*n* = 94) of our isolates. In addition, 51 (19.5%) *C. jejuni* strains representing 27 (32.9%) STs were reported for the first time in the PubMLST database (http://pubmlst.org/campylobacter). The highest Czekanowski index or proportional similarity index (PSI) was calculated for *C. jejuni* strains isolated from human campylobacteriosis cases and broiler products (PSI = 0.32) suggesting a strong link between broiler strains and human cases. The PSI of dairy cattle and human samples was lower (PSI = 0.11), suggesting a weaker link between bovine strains and human cases. The calculated Simpson’s index of all *C. jejuni* isolates showed a high genetic diversity (D = 0.96).

**Conclusion:**

Our results suggest that broiler products are the most important source of human campylobacteriosis in Lithuania. The study provides information on MLST type distribution and genetic relatedness of *C. jejuni* strains from humans, broiler products and dairy cattle in Lithuania for the first time, enabling a better understanding of the transmission pathways of *C. jejuni* in this country.

## Background

Campylobacteriosis is the most commonly reported zoonosis in the European Union (EU) in the last decade, with more than 90% of infections caused by *Campylobacter* (*C.*) *jejuni* [1]. According to the Lithuanian Center for Communicable Diseases and Acquired Immune Deficiency Syndrome (AIDS) the incidence of human *Campylobacter* infections in Lithuania increased from 0.8 to 42.7 cases per 100.000 population during the period of 2003–2016, resulting in 1.225 cases in the year 2016.

In most countries, the main sources of human campylobacteriosis are contaminated poultry products [1–3]. However, *C. jejuni* can be detected in other food animals as well [4, 5], with growing evidence that sources other than poultry products, such as cattle, might be a significant reservoir for human infections [6, 7].

Detection of differences in the relative occurrence of bacterial genotypes in different hosts can be used to estimate the proportion of human *Campylobacter* infections attributable to different host sources. Multilocus Sequence Typing (MLST) displays a reasonable level of heterogeneity of *Campylobacter* sequence types (STs) among the different sources and has become one of the most extensively used molecular typing methods for *C. jejuni* population analysis and can be applied for source attribution studies [8–11].

To our knowledge, data on *Campylobacter* epidemiology (based on MLST analysis of strains originating from different sources) are missing in the Baltic region. To fill this gap, the present study was aimed to investigate the phylogenetic relationship among *C. jejuni* strains isolated from human diarrhoeal patients, broiler products (fresh broiler chicken meat) and dairy cattle in Lithuania.

## Methods

### *C. jejuni* strains

A total of 262 *C. jejuni* strains were included in this study. This *C. jejuni* strain collection was composed of 43 strains isolated from dairy cattle at farm level between May 2012 and August 2012, 102 strains isolated from retail broiler products collected between October 2011 and October 2012 and 117 clinical human *C. jejuni* strains that were collected at the Microbiological Laboratory of Kaunas Clinical Hospital between September 2011 and October 2012. All human *Campylobacter* strains were isolated from sporadic cases associated with gastroenteritis. Isolation of *C. jejuni* was performed by bacteriological standard procedures and species were verified by PCR according to Wang et al. (2002) [12]. The isolates were stored at −80 °C.

### DNA isolation

One μl loop of bacterial culture grown on blood agar plates was collected and suspended in 200 μl of PrepMan Ultra (Applied Biosystems, Foster City, USA). The suspension was vortexed for 10–30 s in order to dissolve the bacterial culture and subsequently heated at 100 °C for 10 min for lysis. Afterwards samples were centrifuged at 16000 g for 3 min. The supernatant containing bacterial DNA was used immediately or transferred to a new tube and stored at −20 °C until use.

### Genotyping of *C. jejuni*

MLST was carried out as described by Dingle et al. (2001) [8]. Amplifications of the seven housekeeping genes that are included in the MLST scheme (*aspA, glnA, gltA, glyA, pgm, tkt, uncA*) were performed in separate tubes in a final volume of 25 μl PCR reaction mix composed of 12.5 μl DreamTaqGreen PCR Master Mix (2X) (Thermo Scientific, Waltham, USA), 8.1 μl Milli-Q water, 1 μmol l^−1^ (2.5 μl) of each forward and reverse primer mix and 2 μl of *C. jejuni* DNA (~40 ng). The amplified PCR products were purified with Thermo Scientific GeneJET PCR Purification Kit (Thermo Scientific). For the sequencing reaction, Thermo-Fast 96-well non-skirted plates were used in combination with adhesive PCR films. Each sequencing reaction was performed in a final volume of 10 μl, using 8.2 μl sequencing mastermix, 0.8 μl primer and 1.0 μl of purified PCR product. The sequencing mastermix contained 1.0 μl BigDye v1.1, 2.0 μl 5X BigDye sequencing buffer (both Thermo Fisher) and 5.2 μl ddH_2_O. All of the used primers were diluted to 5 μmol l^−1^ concentrations. Amplification of samples was carried out in a thermocycler using 25 cycles (denaturation at 96 °C 0.1 min, annealing at 50 °C for 0.05 min, and extension at 60 °C for 1.0 min). After this step, products were purified. To each well of the amplified products 45 μl SAMTM solution and 10 μl XTerminator solution (both Thermo Fisher) was added. The reaction plates were vortexed for 30 min at 1800 rpm and then centrifuged at 1000 x g for 2 min. Sequencing was done on an ABI 3500XL automated DNA sequencer in both orientations.

The obtained sequencing data were imported, checked for quality and analyzed with the BioNumerics v 7.1 software (Applied Maths, Sint-Martens-Latem, Belgium). Allele numbers for each housekeeping gene, sequence types (STs), and clonal complexes (CCs) were assigned by submitting the DNA sequences to the *C. jejuni* MLST database (http://pubmlst.org/campylobacter).

### Statistical analysis

Phylogenetic relationships of the isolates were calculated by MLST-based cluster analysis in Bionumerics using concatenated sequences. The Simpson’s index (D) described by Hunter (1990) [13] was used to determine the genetic diversity of *C. jejuni* genotypes:

$$ D=1-\frac{1}{N\left( N-1\right)}\sum_{j=1}^s nj\left( nj-1\right) $$

N - number of isolates tested;

S - number of different genotypes;

nj - number of isolates belonging to type j.

The Czekanowski index or proportional similarity index (PSI) was calculated to compare STs proportional distribution among *C. jejuni* isolates from various sources [14, 15]. The PSI was calculated by:

$$ PSI=1-0.5{\sum}_i\left|{p}_i-{q}_i\right|={\sum}_i\mathit{\min}\left({p}_i,{q}_i\right) $$

*p*_*i*_ and *q*_*i*_ represent the proportion of strains belonging to ST *i* out of all strains typed from sources *p* and *q*. The values for PSI range between 1 for identical frequency distributions, to 0 for distributions with no common types.

## Results

### Overall genetic diversity of *C. jejuni* isolates

Among the 262 *C. jejuni* strains included in the MLST analysis, 82 distinct sequence types (STs) were identified. Theses STs were assigned to 22 previously described clonal complexes (CCs) (Table 1). Forty (15.3%) strains were assigned to 19 STs which did not match any of the CCs of the MLST database. In addition, 51 (19.5%) *C. jejuni* strains representing 27 (32.9%) STs were previously unreported in the PubMLST database (http://pubmlst.org/campylobacter). The calculated Simpson’s index of all *C. jejuni* isolates showed a high genetic diversity (D = 0.96).

**Table 1:** Distribution of *C. jejuni* MLST genotypes numbers obtained from different sources

Source	No. of strains typed by MLST	No. of STs	No. of CCs	No. of STs not assigned to CCs	No. of new STs	No. of new alleles
Human	117	45	19	3	8	-
Broiler products	102	46	16	12	19	2
Dairy cattle	43	16	9	4	3	1
Total	262	82	22	19	27	3

**Fig. 1:**
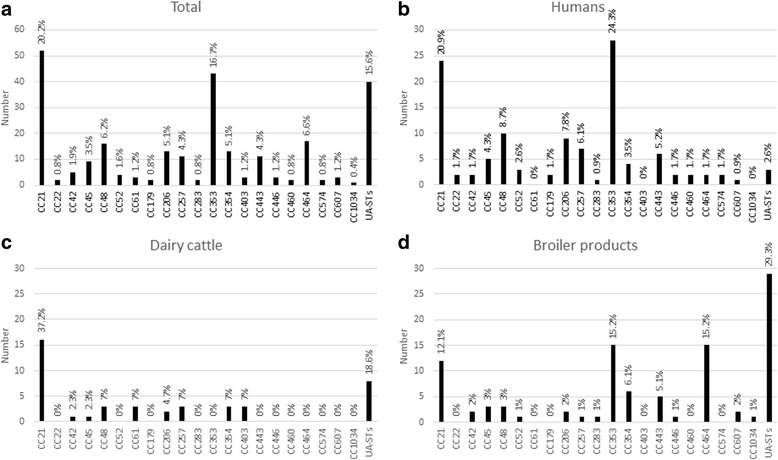
Distribution of clonal complexes (CCs) of *C. jejuni* isolates (**a** All isolates, **b** Human isolates, **c** Dairy cattle isolates, **d** Broiler product isolates) (UA-STs STs not assigned to clonal complexes)

Out of the 22 clonal complexes identified, eight CCs dominated (CC21, CC353, CC464, CC48, CC206, CC354, CC257 and CC443) (Fig. 1a) and 176 (67.2%) of our isolates were assigned to these clonal complexes (Table 2).

**Table 2:** Diversity of clonal complexes (CCs) and sequence types (STs) of *C. jejuni* isolates from human clinical cases, dairy cattle and broiler products

CC	ST	Children	Adults	Dairy cattle	Broiler products	Total	aspA	glnA	gltA	glyA	pgm	tkt	uncA
CC21	19	5	1	1	1	8	2	1	5	3	2	1	5
	21	2		14	2	18	2	1	1	3	2	1	5
	44		1			1	8	1	6	3	2	1	1
	50	11			3	14	2	1	12	3	2	1	5
	251				1	1	2	1	5	2	2	1	5
	376	1			3	4	2	2	1	3	2	1	5
	1459	1				1	2	1	1	2	2	1	5
	1943	1				1	2	1	1	3	2	226	5
	**6393**	1				1	2	1	5	3	11	1	5
	**6436**				1	1	2	1	12	3	2	1	**386**
	**7211**				1	1	2	1	5	3	2	3	26
	**7216**			1		1	2	1	209	3	2	1	5
	Total	22	2	16	12	52							
CC22	22	1				1	1	3	6	4	3	3	3
	1947	1				1	1	94	6	4	3	3	3
	Total	2				2							
CC42	42	2		1	2	5	1	2	3	4	5	9	3
	Total	2		1	2	5							
CC45	45	1				1	4	7	10	4	1	7	1
	137		3		1	4	4	7	10	4	42	7	1
	233				2	2	2	7	10	4	1	7	1
	583	1				1	4	7	10	4	42	51	1
	**7318**			1		1	4	7	10	**632**	42	51	1
	Total	2	3	1	3	9							
CC48	38			3		3	2	4	2	2	6	1	5
	429	5			1	6	7	4	5	2	11	1	5
	475				1	1	2	4	1	4	19	62	5
	918	2	3		1	6	2	4	1	4	19	1	5
	Total	7	3	3	3	16							
CC52	2066	3			1	4	9	10	5	10	22	3	6
	Total	3			1	4							
CC61	61			3		3	1	4	2	2	6	3	17
	Total			3		3							
CC179	2209	2				2	1	6	29	176	40	32	3
	Total	2				2							
CC206	122	1				1	6	4	5	2	2	1	5
	227	5	2		2	9	2	4	5	2	2	1	5
	572		1	2		3	62	4	5	2	2	1	5
	Total	6	3	2	2	13							
CC257	257	5		3		8	9	2	4	62	4	5	6
	824	2			1	3	9	2	2	2	11	5	6
	Total	7		3	1	11							
CC283	**6382**	1				1	4	2	40	4	42	51	1
	**7210**				1	1	2	7	40	4	2	51	5
	Total	1			1	2							
CC353	5	23	1		9	33	7	2	5	2	10	3	6
	353	2			2	4	7	17	5	2	10	3	6
	356	1				1	14	17	5	2	11	3	6
	3285	1				1	7	2	5	2	10	3	1
	5011				1	1	7	84	5	2	19	3	26
	**6413**				1	1	7	17	2	15	2	3	6
	**6435**				1	1	7	114	5	2	67	**520**	6
	**7212**				1	1	7	84	5	2	19	67	6
	Total	27	1		15	43							
CC354	354	1	1		3	5	8	10	2	2	11	12	6
	**6381**		1			1	73	17	2	2	11	12	6
	**6466**	1				1	14	2	2	2	11	12	6
	6784				1	1	8	10	2	2	11	5	6
	**7215**			3	1	4	14	10	2	2	11	12	6
	**7309**				1	1	1	71	2	2	11	67	6
	Total	2	2	3	6	13							
CC403	1775			1		1	10	27	59	19	10	5	7
	933			2		2	10	1	59	19	10	5	7
	Total			3		3							
CC443	51	3	2			5	7	17	2	15	23	3	12
	**6391**	1			4	5	4	17	2	15	2	3	12
	**7208**				1	1	7	17	2	15	23	3	6
	Total	4	2		5	11							
CC446	446	1				1	47	55	5	10	11	68	8
	**6392**	1			1	2	47	55	2	10	11	68	8
	Total	2			1	3							
CC460	670	1				1	77	30	2	2	89	59	6
	**6467**	1				1	77	30	2	2	11	59	6
	Total	2				2							
CC464	464	2			15	17	24	2	2	2	10	3	1
	Total	2			15	17							
CC574	305	2				2	9	53	2	10	11	3	3
	Total	2				2							
CC607	607	1			2	3	8	2	5	53	11	3	1
	Total	1			2	3							
CC658	658	1			3	4	2	4	2	4	19	3	6
	**6468**	1				1	2	4	12	93	11	3	6
	Total	2			3	5							
CC1034	**6409**				1	1	2	15	4	3	154	25	23
	Total				1	1							
	436			1		1	7	21	5	62	4	61	44
	495	1				1	67	2	42	4	4	25	8
	1721	1				1	7	78	42	4	106	12	8
	2217			1		1	19	24	42	246	300	259	57
	2883				1	1	7	21	5	62	67	3	216
	3098			5		5	19	24	23	20	397	16	15
	3502				1	1	18	33	72	325	116	104	6
	3546	1				1	9	17	5	10	350	335	3
	3573				1	1	7	172	21	49	125	224	51
	4800				2	2	275	84	5	10	119	178	26
	5590			1		1	1	2	42	450	10	9	59
	**6410**				12	12	7	112	5	15	119	67	6
	**6411**				6	6	1	61	4	38	517	59	35
	**6412**				1	1	7	112	5	1	13	1	26
	7052				1	1	1	61	4	38	517	59	6
	**7207**				1	1	9	61	2	2	517	5	35
	**7209**				1	1	7	112	5	15	42	67	1
	**7213**				1	1	7	112	5	15	119	12	6
	**7308**				1	1	275	84	5	10	19	178	26
	Total	3		8	29	40							
Total		101	16	43	102	262							

(Novel STs and alleles are indicated in bold)

On ST-level, five sequence types (ST-5, ST-21, ST-464, ST-50 and ST-6410) were dominating and they accounted for 35.9% (*n* = 94) of our isolates (Table 2).

**Fig. 2:**
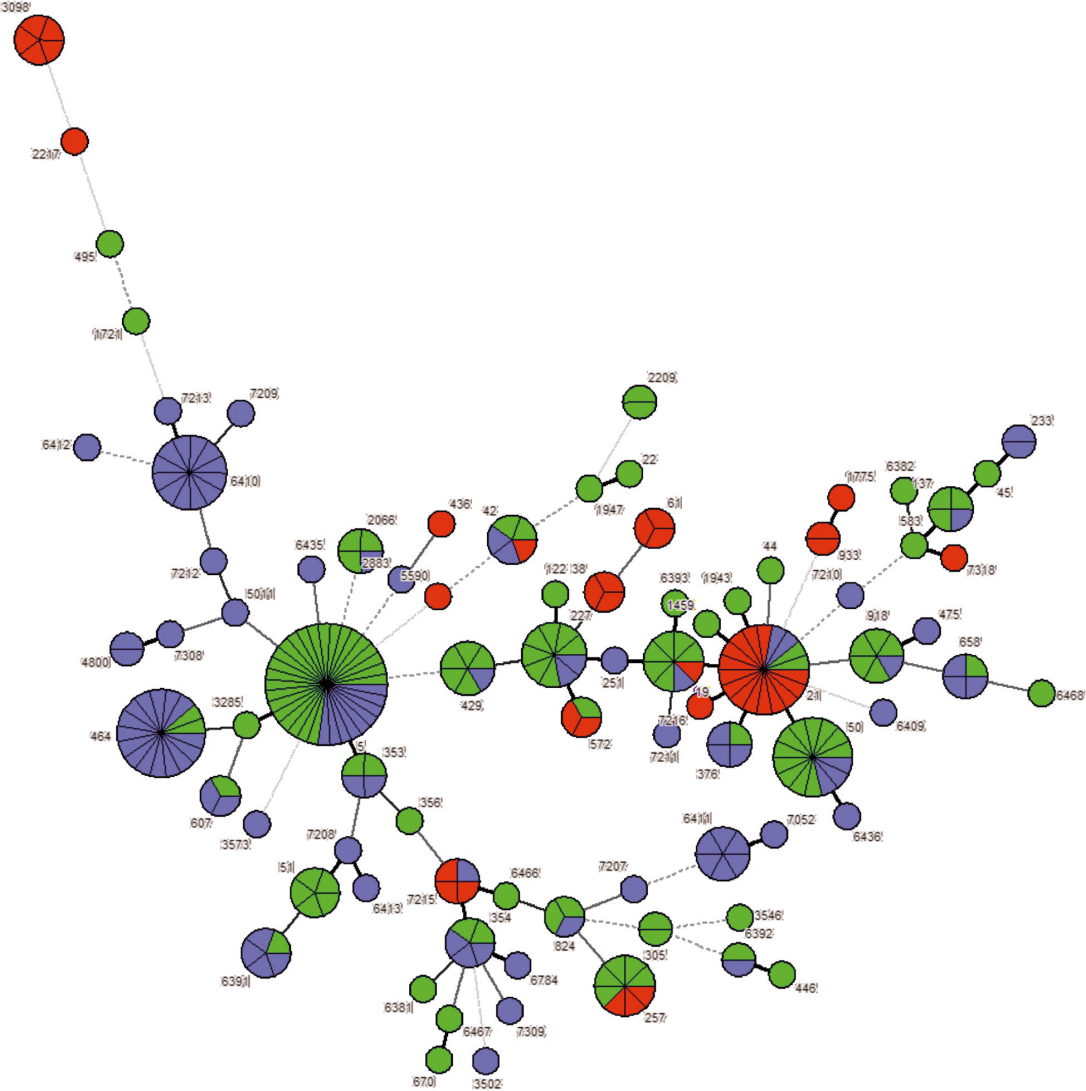
Phylogenetic analysis of *C. jejuni* strains isolated from infected human, broiler products and dairy cattle by MLST (each circle and number by the circle represents one ST; circles of increased diameter represent higher strain numbers within one ST; different colour indicate individual strain source (green-human, violet-broiler products and red-dairy cattle); the thickness and the dotting of the lines indicate the distance between the circles. A thicker line denotes closer distance than a thin line and a thin line denotes closer distance than a dotted line). Minimum spanning tree (MST) analysis carried out by BioNumerics v7.1

A minimal spanning tree was generated from the MLST data illustrating the phylogenetic relationships of the *C. jejuni* strains (Fig. 2). Two STs (ST-257 and ST-572) were identified only from dairy cattle and human clinical cases and 16 STs were identified only from broiler products and human clinical cases. Three sequence types (ST-19, ST-21 and ST-42) were identified in all three sources (i.e. human clinical samples, dairy cattle samples, broiler product samples) (Table 2).

The PSI was calculated to evaluate the proportional similarity and genetic relatedness of sequence types of human isolates and strains isolated from the different tested sources. This analysis showed that STs from broiler product isolates had the highest proportional similarity to STs from human clinical isolates (PSI = 0.32) (Table 3).

**Table 3:** The Simpson’s index (D) and proportional similarity index (PSI) of *C. jejuni* isolates from different sources

Source	D	PSI		
		Broiler products	Dairy cattle	Human
Broiler products	0.95	1		
Dairy cattle	0.87	0.06	1	
Human	0.94	0.32	0.11	1

1 = maximal similarity; 0 = maximal difference

### Genetic diversity of *C. jejuni* strains isolated from humans

Among the 117 human isolates included in the study, 45 different STs were found and eight of them are reported for the first time (Table 1). Out of the 19 identified CCs, three CCs (CC21, CC353 and CC48) were dominant with 62 (53%) of our isolates attributable to these CCs (Fig. 1b). ST-5 and ST-50, which are linked to CC353 and CC21, were predominant in the pool of human isolates and they represent 20.5% and 9.4% of *C. jejuni* isolates from human clinical cases, respectively and these two CCs are genetically related to broiler products. The calculated Simpson’s index of human *C. jejuni* isolates showed a high genetic diversity (D = 0.94) (Table 3). Eight *C. jejuni* isolates from human samples were assigned to novel STs (Table 2). Two of these new STs (ST-6391 and ST-6392) were identified in *C. jejuni* isolates from broiler products as well (Table 2).

### Genetic diversity of *C. jejuni* strains isolated from dairy cattle

Forty-three *C. jejuni* strains from dairy cattle were included in the study and 16 different STs were found (Table 1). Twelve STs representing 35 isolates (81.4%) were assigned to nine previously described CCs. The remaining eight isolates (18.6%) were assigned to four different STs, which could not be assigned to any of the described CCs of PubMLST database. CC21 and ST-21 were dominant among *C. jejuni* isolates detected in dairy cattle, with 37.2% and 32.6% respectively (Fig. 1c, Table 2). Five *C. jejuni* isolates from dairy cattle were assigned to three novel STs (ST-7215, ST-7216 and ST-7318) (Table 2). ST-7215 was identified among *C. jejuni* isolates from broiler products as well (Fig. 1). The lowest diversity of the genotypes was identified among isolates from dairy cattle (D = 0.87) (Table 3).

### Genetic diversity of *C. jejuni* strains isolated from broiler products

Among the 102 *C. jejuni* strains isolated from broiler products, 46 distinct STs were identified (Table 1). These STs were assigned to 16 previously described CCs. Three CCs (CC21, CC353 and CC464) were predominating among *C. jejuni* isolates detected in broiler products and 41.2% of the strains were assigned to one of these three CCs (Fig. 1d). Three STs (ST-5, ST-464 and ST-6410) were dominating (with 8.8%, 14.7% and 11.8%, respectively) among *C. jejuni* isolates detected in broiler products (Table 2). Interestingly, ST-6410, which has not been described before, was one of the most prevalent STs among broiler products. The highest diversity of *C. jejuni* genotypes was found among isolates detected in broiler products (D = 0.95) (Table 3).

## Discussion

In countries like Lithuania, with limited information on the epidemiology and transmission pathways of *Campylobacter*, the genetic characterization and comparison of genotypes of *Campylobacter* isolates from multiple sources is necessary to improve the understanding of *Campylobacter* epidemiology and transmission pathways with focus on the importance of different sources for human cases. By weighing the importance of different sources for human infections, intervention measures can be focused accordingly.

The genetic diversity of *C. jejuni* in dairy cattle and broiler products was investigated and strains from these sources were compared to isolates from human clinical cases. To our knowledge, this is the first study providing information on MLST type distribution and genetic relatedness of *C. jejuni* strains from humans, broiler products and dairy cattle in Lithuania.

By applying MLST analysis, which is widely used for genotyping of *Campylobacter*, a high genetic diversity of *C. jejuni* strains was identified, as widely documented in other countries [2, 3, 16–18]. By MLST analysis, the 262 *C. jejuni* isolates were assigned to 82 STs. However, 27 STs and three novel alleles (glyA-632 in a dairy cattle isolate; tkt-520 and uncA-386 in an isolate from broiler products) were previously unreported in the PubMLST database. As reported by other authors, novel genotypes may represent local clones restricted to a given country [19]. However, more detailed analyses are needed to assess this possibility for Lithuanian or Baltic strains.

Overall, our results suggest that broiler products are the most important source of human campylobacteriosis in Lithuania. The highest PSI was calculated for *C. jejuni* strains isolated from human campylobacteriosis cases and broiler products (PSI = 0.32). Accordingly, Kittl et al. (2013) [3] investigated the PSI for MLST types between *C. jejuni* from different sources and demonstrated that the greatest similarity was observed between human and chicken isolates as well (human-chicken: PSI = 0.61; human-dog: PSI = 0.46). Compared to the link of broiler and human isolates, the PSI of dairy cattle and human samples was lower (PSI = 0.11), suggesting a weaker link between bovine strains and human cases in our study (Table 3). However, the difference between the number of isolates of the individual sources might have biased these results.

Clonal complexes CC21 and CC353 were predominant among *C. jejuni* strains in our study, representing 19.8% and 16.4% of all strains, respectively (Fig. 1a). The highest proportion (44.4%) of human *C. jejuni* isolates was assigned to clonal complexes CC353 and CC21. This is in agreement with different studies demonstrating that CC21 is dominant among the *C. jejuni* population in various geographic regions [8, 17, 20–22]. All human infections included in our study were locally acquired, thus not associated with travel. CC21 is often associated with poultry, cattle, wild birds, sheep and water [17, 21, 23, 24]. In our study, CC21 was dominant among dairy cattle isolates. Within this CC21, ST-21 dominated in dairy cattle but not in broiler products and human isolates. In contrast to these findings, other authors showed that ST-21 is one of the most widely distributed STs among human and broiler *C. jejuni* isolates [25, 26].

The second clonal complex (CC353) that was associated with strains of human campylobacteriosis cases in our study is rather uncommon in Europe [20, 27, 28]. However, CC353 is highly prevalent among e. g. poultry in China and other countries [29, 30].

When looking at ST-level, ST-6410 was identified as a new sequence type in our study. This ST was predominant among broiler products (11.8% of all isolates). However, this ST was not found among dairy cattle isolates and human clinical isolates. ST-464 was dominant among broiler products, representing 14.7% of the isolates, however only two *C. jejuni* isolates from clinical cases were assigned to this ST in our study. Nevertheless, ST-464 is frequently associated with both broiler and human isolates according to PubMLST database and individual studies [3, 31]. Three STs (ST-19, ST-21 and ST-42), identified among dairy cattle and broiler products, were also detected in strains isolated from human clinical cases (Fig. 1). These data showed that *C. jejuni* genotypes have a cross distribution among different sources. Two STs (ST-257 and ST-572) were found only among *C. jejuni* isolates from dairy cattle and human clinical cases and 16 sequence types were identified among *C. jejuni* isolates from broiler products and human (Table 2) highlighting a possible link between individual sources and human infections.

In contrast, a number of CCs and STs were only identified in a single source. Four CCs (CC22, CC179, CC460 and CC574) and 24 STs (Table 2) were identified only among *C. jejuni* isolates from clinical cases of human. One CC1034 and 26 STs were found only among *C. jejuni* isolates from broiler products. Two CCs (CC61 and CC403) and 10 sequence types were identified only among *C. jejuni* isolates from dairy cattle. These data suggest that some genotypes might be restricted to a specific source. However further analyses are needed to confirm this hypothesis.

## Conclusions

In conclusion, the identified overlap of *C. jejuni* genotypes isolated from human and broiler products or (to a lesser degree) from humans and dairy cattle highlights the importance of these sources for human campylobacteriosis in Lithuania. 74.4% of all *C. jejuni* isolates from human clinical cases were assigned to STs which were identified among *C. jejuni* isolates from broiler products and dairy cattle.

## Abbreviations

CC: Clonal complex
D: The Simpson’s index
DNA: Deoxyribonucleic acid
EU: European Union
MLST: Multilocus sequence typing
MST: Minimum spanning tree
PCR: Polymerase chain reaction
PSI: Proportional similarity index or Czekanowski index
ST: Sequence type

## References

[1] European Food Safety Authority, European Centre for Disease Prevention and Control. The European Union Summary Report on Trends and Sources of Zoonoses, Zoonotic Agents and Food-borne Outbreaks in 2013. EFSA Journal. 2015;13(1):3991.

[2] Sheppard SK, Dallas JF, Strachan NJ, MacRae M, McCarthy ND, Wilson DJ (2009). *Campylobacter* genotyping to determine the source of human infection. Clin Infect Dis.

[3] Kittl S, Heckel G, Korczak BM, Kuhnert P (2013). Source attribution of human *Campylobacter* isolates by MLST and fla-typing and association of genotypes with quinolone resistance. PLoS One.

[4] Dasti JI, Tareen AM, Lugert R, Zautner AE, Gross U (2010). *Campylobacter jejuni*: a brief overview on pathogenicity-associated factors and disease-mediating mechanisms. Int J Med Microbiol.

[5] Vasiliki I, Anastasios I, Emmanouil M, Pantelis B, Chryssoula N, Nicolaos L (2013). Multilocus sequence typing (and phylogenetic analysis) of *Campylobacter jejuni* and *Campylobacter coli* strains isolated from clinical cases in Greece. BMC Res Notes.

[6] Mughini Gras L, Smid JH, Wagenaar JA, de Boer AG, Havelaar AH, Friesema IH (2012). Risk factors for campylobacteriosis of chicken, ruminant, and environmental origin: a combined case-control and source attribution analysis. PLoS One.

[7] Levesque S, Fournier E, Carrier N, Frost E, Arbeit RD, Michaud S (2013). Campylobacteriosis in urban versus rural areas: a case-case study integrated with molecular typing to validate risk factors and to attribute sources of infection. PLoS One.

[8] Dingle KE, Colles FM, Wareing DR, Ure R, Fox AJ, Bolton FE (2001). Multilocus sequence typing system for *Campylobacter jejuni*. J Clin Microbiol.

[9] Mullner P, Spencer SE, Wilson DJ, Jones G, Noble AD, Midwinter AC (2009). Assigning the source of human campylobacteriosis in New Zealand: a comparative genetic and epidemiological approach. Infect Genet Evol.

[10] Strachan NJ, Gormley FJ, Rotariu O, Ogden ID, Miller G, Dunn GM (2009). Attribution of *Campylobacter* infections in northeast Scotland to specific sources by use of multilocus sequence typing. J Infect Dis.

[11] Colles FM, Maiden MC (2012). *Campylobacter* sequence typing databases: applications and future prospects. Microbiol.

[12] Wang G, Clark CG, Taylor TM, Pucknell C, Barton C, Price L (2002). Colony multiplex PCR assay for identification and differentiation of *Campylobacter jejuni*, *C. coli*, *C. lari*, *C. upsaliensis* and *C. fetus* subsp. *fetus*. J Clin Microbiol.

[13] Hunter P (1990). Reproducibility and indices of discriminatory power of microbial typing methods. J Clin Microbiol.

[14] Feinsinger P, Spears EE, Poole RW (1981). A simple measure of niche breadth. Ecology.

[15] Rosef O, Kapperud G, Lauwers S, Gondrosen B (1985). Serotyping of *Campylobacter jejuni*, *Campylobacter coli*, and *Campylobacter laridis* from domestic and wild animals. Appl Environ Microbiol.

[16] Dingle KE, Colles FM, Falush D, Maiden MC (2005). Sequence typing and comparison of population biology of *Campylobacter coli* and *Campylobacter jejuni*. J Clin Microbiol.

[17] de Haan CP, Kivisto R, Hakkinen M, Rautelin H, Hanninen ML (2010). Decreasing trend of overlapping multilocus sequence types between human and chicken *Campylobacter jejuni* isolates over a decade in Finland. Appl Environ Microbiol.

[18] Griekspoor P, Engvall EO, Olsen B, Waldenstrom J (2010). Multilocus sequence typing of *Campylobacter jejuni* from broilers. Vet Microbiol.

[19] Stone D, Davis M, Baker K, Besser T, Roopnarine R. Sharma R. MLST genotypes and antibiotic resistance of *Campylobacter* spp isolated from poultry in Grenada Biomed Res Int. 2013;2013:794643. doi:10.1155/2013/794643. 10.1155/2013/794643 PMC3595693 23555097

[20] Dingle KE, Colles FM, Ure R, Wagenaar JA, Duim B, Bolton FJ (2002). Molecular characterization of *Campylobacter jejuni* clones: a basis for epidemiologic investigation. Emerg Infect Dis.

[21] Colles FM, Jones K, Harding RM, Maiden MC (2003). Genetic diversity of *Campylobacter jejuni* isolates from farm animals and the farm environment. Appl Environ Microbiol.

[22] Smid JH, Mughini Gras L, de Boer AG, French NP, Havelaar AH, Wagenaar JA (2013). Practicalities of using non-local or non-recent multilocus sequence typing data for source attribution in space and time of human campylobacteriosis. PLoS One.

[23] Sopwith W, Birtles A, Matthews M, Fox A, Gee S, Painter M (2008). Identification of potential environmentally adapted *Campylobacter jejuni* strain. United Kingdom Emerg Infect Dis.

[24] Magnusson SH, Guðmundsdottir S, Reynisson E, Runarsson AR, Harðardottir H, Gunnarson E (2011). Comparison of *Campylobacter jejuni* isolates from human, food, veterinary and environmental sources in Iceland using PFGE, MLST and fla-SVR sequencing. J Appl Microbiol.

[25] Ragimbeau C, Schneider F, Losch S, Even J, Mossong J (2008). Multilocus sequence typing, pulsed-field gel electrophoresis, and fla short variable region typing of clonal complexes of *Campylobacter jejuni* strains of human, bovine, and poultry origins in Luxembourg. Appl Environ Microbiol.

[26] Harvala H, Rosendal T, Lahti E, Engvall EO, Brytting M, Wallensten A, Lindberg A. Epidemiology of *Campylobacter jejuni* infections in Sweden, November 2011–October 2012: is the severity of infection associated with *C. jejuni* sequence type? Infect Ecol Epidemiol. 2016;6:31079. doi: 10.3402/iee.v6.31079. 10.3402/iee.v6.31079 PMC4826459 27059819

[27] Duim B, Godschalk PC, van den Braak N, Dingle KE, Dijkstra JR, Leyde E (2003). Molecular evidence for dissemination of unique *Campylobacter jejuni* clones in curacao. Netherlands Antilles J Clin Microbiol.

[28] Manning G, Dowson CG, Bagnall MC, Ahmed IH, West M, Newell DG (2003). Multilocus sequence typing for comparison of veterinary and human isolates of *Campylobacter jejuni*. Appl Environ Microbiol.

[29] Kinana AD, Cardinale E, Tall F, Bahsoun I, Sire JM, Garin B (2006). Genetic diversity and quinolone resistance in *Campylobacter jejuni* isolates from poultry in Senegal. Appl Environ Microbiol.

[30] Zhang M, Gu Y, He L, Ran L, Xia S, Han X (2010). Molecular typing and antimicrobial susceptibility profiles of *Campylobacter jejuni* isolates from north China. J Med Microbiol.

[31] Cody AJ, McCarthy NM, Wimalarathna HL, Colles FM, Clark L, Bowler IC (2012). A longitudinal 6-year study of the molecular epidemiology of clinical *Campylobacter* isolates in Oxfordshire. United Kingdom J Clin Microbiol.

